# Tag SNPs in long non-coding RNA H19 contribute to susceptibility to gastric cancer in the Chinese Han population

**DOI:** 10.18632/oncotarget.3840

**Published:** 2015-04-15

**Authors:** Chao Yang, Ran Tang, Xiang Ma, Younan Wang, Dakui Luo, Zekuan Xu, Yi Zhu, Li Yang

**Affiliations:** ^1^ Department of General Surgery, The First Affiliated Hospital of Nanjing Medical University, Nanjing, China; ^2^ Jiangsu Province Academy of Clinical Medicine, Institute of Tumor Biology, Nanjing, China

**Keywords:** gastric cancer, H19, polymorphism, genotype

## Abstract

Long non-coding RNA (lncRNA) H19 is involved in tumor development, progression, and metastasis. This case-control study assessed the association between H19 genetic variants and susceptibility to gastric cancer (GC) in a Chinese Han population. We genotyped four lncRNA H19 single nucleotide polymorphisms (SNPs) (rs217727 C > T, rs2839698 C > T, rs3741216 A > T, rs3741219 T > C) in 500 GC patients and 500 healthy controls. Carriers of variant rs217727T and rs2839698T alleles showed increased GC risk (*P* = 0.008 and 0.011, respectively). Compared with the common genotype, CT + TT rs217727 and CT + TT rs2839698 genotypes were associated with significantly increased GC risk (*P* = 0.040, adjusted odds ratio [OR] = 1.32, 95% confidence interval [CI] = 1.01–1.71; *P* = 0.033, adjusted OR = 1.31, 95% CI = 1.02–1.69, respectively). Further stratified analyses revealed that the association between GC risk and variant genotypes of rs217727 was more profound in younger individuals (≤59 years) and non-smokers, while the association between risk and the rare rs2839698 genotype persisted in men and rural subjects. rs2839698 CT and TT genotypes were also associated with higher serum H19 mRNA levels compared with the CC genotype. These findings suggest that lncRNA H19 SNPs may contribute to susceptibility to GC.

## INTRODUCTION

Gastric cancer (GC) ranks fourth in terms of incidence and second in terms of mortality among all cancers worldwide [1, 2]. Despite the decrease in incidence in some regions of the world, GC continues to present a major clinical challenge, because most cases are diagnosed at advanced stages, with consequently poor prognosis and limited treatment options. The development of GC is a complex and multifactorial process involving a number of etiological factors and multiple genetic and epigenetic alterations [3, 4]. *Helicobacter pylori* infection, high salt intake, and tobacco smoking are the main environmental factors influencing its development [2], though genetic factors are also thought to play important roles in gastric carcinogenesis [5]. Previous epidemiologic studies provided evidence to support an association between genetic polymorphisms and the risk of GC [6-9].

Various long, non-coding (lnc) RNAs have recently been implicated in many human diseases, including cancers, and emerging studies are beginning to unravel the molecular mechanisms underlying lncRNA function in these pathological processes [10-13]. lncRNA H19 is an imprinted gene located within the highly-conserved imprinted H19/insulin-like growth factor 2 (IGF2) locus on chromosome 11p15.5. Methylation of CpG islands in the H19 5′-flanking region, the so-called differentially-methylated region (DMR), is critical for the regulation of H19 and IGF2 gene expression [14, 15]. Binding of the CCCTC-binding factor insulator protein to the non-methylated allele of the DMR promotes H19 expression, whereas IGF2 is expressed from the methylated allele [15].

The human H19 gene encodes a 2.3-kb long, spliced, poly-adenylated non-coding RNA that plays important roles in embryonic development and growth control [16-19]. However, H19 expression is reduced after birth, and its expression is only found in cardiac and skeletal muscle [20]. Increasing evidence suggests that H19 is abnormally expressed in breast, liver, lung, cervical, esophageal, and bladder tumors [21-26], and promotes cancer cell proliferation, suggesting an oncogenic function. However, no studies to date have reported on the association between genetic variants of H19 and the risk of malignant diseases, including GC.

In this study, we hypothesized that single nucleotide polymorphisms (SNPs) in lncRNAs may be associated with the risk of GC. To test this hypothesis, we genotyped four H19 lncRNA tag SNPs (rs217727, rs2839698, rs3741216, and rs3741219) in a case-control study of 500 patients with GC and 500 healthy controls from the Chinese Han population.

## RESULTS

### Characteristics of the study population

Overall, 500 patients with GC and 500 healthy controls were enrolled in this study. The characteristics of the cases and controls are summarized in Table 1. There were no significant differences between the cases and controls in the frequency distributions of age and sex (*P* = 0.575 and 0.127), suggesting successful matching of subjects. The mean age (SD) of GC patients (289 men and 211 women) was 58.7 (10.7) years and that of the healthy controls (265 men and 235 women) was 59.2 (13.5) years. There were no significant differences between the cases and controls in terms of history of hypertension, diabetes, smoking and residence.

**Table 1:** Demographic information

Characteristics	Cases (n = 500)	Controls (n = 500)	*P* value
Age (years, mean±SD)	58.7±10.7	59.2±13.5	0.575
Sex, n (%)			
Female	211 (42.2)	235 (47.0)	
Male	289 (57.8)	265 (53.0)	0.127
Hypertension, n (%)			
No	371 (74.2)	362 (72.4)	
Yes	129 (25.8)	138 (27.6)	0.520
Diabetes, n (%)			
No	449 (89.8)	439 (87.8)	
Yes	51 (10.2)	61 (12.2)	0.316
Smoking, n (%)			
Never	376 (75.2)	359 (71.8)	
Ever	124 (24.8)	141 (28.2)	0.223
Residence, n (%)			
Rural	288 (57.6)	264 (52.8)	
Urban	212 (42.4)	236 (47.2)	0.127
Tumor differentiation, n (%)			
Well	20 (4.0)		
Moderate	123 (24.6)		
Poor	357 (71.4)		
Depth of tumor infiltration, n (%)			
T1	92 (18.4)		
T2	58 (11.6)		
T3	158 (31.6)		
T4	192 (38.4)		
Lymph node metastasis, n (%)			
Negative	170 (34.0)		
Positive	330 (66.0)		
Localization, n (%)			
Cardia	221 (44.2)		
Noncardia	279 (55.8)		

SD standard deviation

### H19 variants and risk of GC

The genotype and allele frequencies of the selected SNPs and their associations with risk of GC are summarized in Table 2. The genotype distributions of the SNPs in the controls were in agreement with the Hardy-Weinberg equilibrium (*P* = 0.296 for rs217727, *P* = 0.175 for rs2839698, *P* = 0.221 for rs3741216 and *P* = 0.245 for rs3741219). The TT genotype and T allele of H19 rs217727 were associated with significantly increased risks of GC compared with the CC genotype and C allele (TT vs. CC: *P* = 0.009, adjusted OR = 1.68, 95% CI = 1.14-2.49; T *vs*. C: *P* = 0.008, adjusted OR = 1.27, 95% CI = 1.06-1.52). Compared with individuals with the wild-type CC genotype, subjects with the variant genotypes (CT+TT) had a significantly increased risk of GC (*P* = 0.040, adjusted OR = 1.32, 95% CI = 1.01-1.71). The variant TT and (CT+TT) genotypes and T allele of rs2839698 were associated with a significantly increased risk of GC, compared with the CC genotype and C allele (TT vs. CC: *P* = 0.024, adjusted OR = 1.68, 95% CI = 1.07-2.63; CT+TT *vs*. CC: *P* = 0.033, adjusted OR = 1.31, 95% CI = 1.02-1.69; T *vs*. C: *P* = 0.011, adjusted OR = 1.29, 95% CI = 1.06-1.57, respectively). However, there were no significant associations between the genotypes of the other two H19 SNPs (rs3741216 A > T and rs3741219 T > C) and risk of GC.

**Table 2:** Genotype and allele frequencies of H19 SNPs in cases and controls, and genotype- and allelotype-specific risks

genotype	Cases (n = 500) (%)	Controls (n = 500) (%)	Crude OR (95% CI)	P value	Adjusted OR (95% CI)^a^	*P* value
rs217727						
CC	160 (32.0)	193 (38.6)	1.00		1.00	
CT	252 (50.4)	244 (48.8)	1.25 (0.95-1.64)	0.116	1.22 (0.93-1.61)	0.155
TT	88 (17.6)	63 (12.6)	**1.69 (1.15-2.48)**	**0.008**	**1.68 (1.14-2.49)**	**0.009**
CT + TT	340 (68.0)	307 (61.4)	**1.34 (1.03-1.73)**	**0.029**	**1.32 (1.01-1.71)**	**0.040**
CC + CT	412 (82.4)	437 (87.4)	1.00		1.00	
TT	88 (17.6)	63 (12.6)	**1.48 (1.04-2.10)**	**0.028**	**1.48 (1.04-2.10)**	**0.030**
C	572 (57.2)	630 (63.0)	1.00			
T	428 (42.8)	370 (37.0)	**1.27 (1.06-1.52)**	**0.008**		
rs2839698						
CC	250 (50.0)	284 (56.8)	1.00		1.00	
CT	195 (39.0)	178 (35.6)	1.24 (0.96-1.62)	0.106	1.24 (0.95-1.62)	0.112
TT	55 (11.0)	38 (7.6)	**1.64 (1.05-2.57)**	**0.029**	**1.68 (1.07-2.63)**	**0.024**
CT + TT	250 (50.0)	216 (43.2)	**1.32 (1.03-1.69)**	**0.031**	**1.31 (1.02-1.69)**	**0.033**
CC + CT	445 (89.0)	462 (92.4)	1.00		1.00	
TT	55 (11.0)	38 (7.6)	1.50 (0.97-2.32)	0.066	1.52 (0.98-2.36)	0.058
C	695 (69.5)	746 (74.6)	1.00			
T	305 (30.5)	254 (25.4)	**1.29 (1.06-1.57)**	**0.011**		
rs3741216						
AA	380 (76.0)	379 (75.8)	1.00		1.00	
AT	102 (20.4)	109 (21.8)	0.93 (0.69-1.27)	0.658	0.95 (0.70-1.30)	0.754
TT	18 (3.6)	12 (2.4)	1.50 (0.71-3.15)	0.289	1.53 (0.72-3.25)	0.266
AT + TT	120 (24.0)	121 (24.2)	0.99 (0.74-1.32)	0.941	1.01 (0.76-1.36)	0.933
AA + AT	482 (96.4)	488 (97.6)	1.00		1.00	
TT	18 (3.6)	12 (2.4)	1.52 (0.72-3.19)	0.269	1.56 (0.74-3.28)	0.246
A	862 (86.2)	867 (86.7)	1.00			
T	138 (13.8)	133 (13.3)	1.04 (0.81-1.35)	0.744		
rs3741219						
TT	260 (52.0)	268 (53.6)	1.00		1.00	
TC	187 (37.4)	189 (37.8)	1.02 (0.78-1.33)	0.884	1.01 (0.77-1.32)	0.958
CC	53 (10.6)	43 (8.6)	1.27 (0.82-1.97)	0.283	1.31 (0.84-2.03)	0.236
TC + CC	240 (48.0)	232 (46.4)	1.07 (0.83-1.37)	0.612	1.06 (0.82-1.36)	0.671
TT + TC	447 (89.4)	457 (91.4)	1.00		1.00	
CC	53 (10.6)	43 (8.6)	1.26 (0.83-1.92)	0.284	1.27 (0.83-1.94)	0.271
T	707 (70.7)	725 (72.5)	1.00			
C	293 (29.3)	275 (27.5)	1.09 (0.90-1.33)	0.372		

The bold in the table indicates statistically significant data.

a Adjusted for age, sex, smoking status, residence, hypertension, and diabetes.

We evaluated the combined effects of the variant alleles of the independent SNPs (H19 rs217727 and rs2839698) on GC risk. The linkage disequilibrium (LD) analysis showed that these two SNPs were not in LD in the controls (D′ = 0.23 and r^2^ = 0.12 for rs217727 C > T and rs2839698 C > T). The ORs for risk of GC development increased with increasing number of variant alleles (Table 3). Subjects carrying three to four variant alleles of rs217727 and rs2839698 were at significantly increased GC risk (*P* < 0.001, adjusted OR = 10.77, 95% CI = 3.90-29.72) compared with subjects wild-type homozygous for the two SNPs.

**Table 3:** Combined favorable alleles (rs217727-T and rs2839698-T) and gastric cancer risk

Variables	Controls n (%)	Cases n (%)	Crude OR (95 % CI)	P value	Adjusted OR (95% CI)^a^	*P* value
0	79 (15.8)	51 (10.2)	1.00		1.00	
1	223 (44.6)	205 (41.0)	**1.42 (0.96-2.12)**	**0.083**	**1.45 (0.97-2.18)**	**0.071**
2	193 (38.6)	210 (42.0)	**1.69 (1.13-2.52)**	**0.011**	**1.61 (1.07-2.43)**	**0.022**
3-4	5 (1.0)	34 (6.8)	10.53 (3.87-28.71)	<0.001	10.77 (3.90-29.72)	<0.001
Trend						
0	79 (15.8)	51 (10.2)	1.00		1.00	
1-4	421 (84.2)	449 (89.8)	**1.65 (1.13-2.41)**	**0.009**	**1.63 (1.12-2.38)**	**0.011**

The bold in the table indicates statistically significant data.

a Adjusted for age, sex, smoking status, residence, hypertension, and diabetes.

### Functional assay for SNP in the promoter region of H19

**Figure 1:**
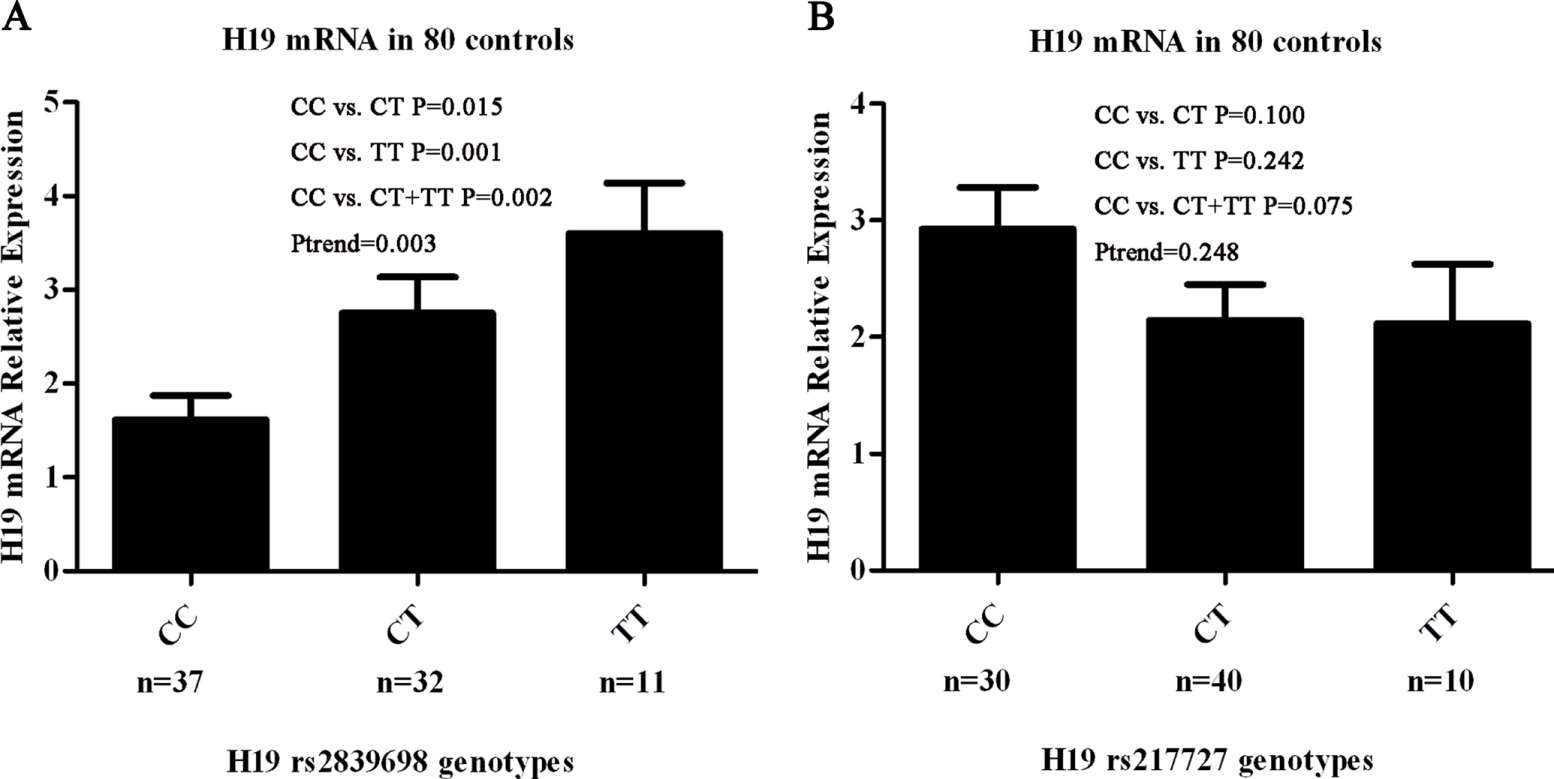
Correlation between rs2839698 and rs217727 genotypes and expression of H19 mRNA **A.** Genotype–phenotype correlation for rs2839698 and relative expression levels of H19 mRNA in serum from 80 cancer-free controls. Relative H19 mRNA expression levels were significantly higher for the CT (2.75 ± 0.38), TT (3.60 ± 0.54) and CT + TT genotypes (2.97 ± 0.32) than the CC genotype (1.61 ± 0.26) (*P* = 0.015, 0.001 and 0.002, respectively). **B.** Genotype–phenotype correlation for rs217727 and relative expression levels of H19 mRNA in serum from 80 cancer-free controls. Relative H19 mRNA expression levels were similar among the three groups with rs217727 CC, CT and TT genotypes (*P* = 0.100 for CC versus CT, *P* = 0.242 for CC versus TT and *P* = 0.075 for CC versus CT/TT).

We further explored the biological significance of H19 rs217727 and rs2839698 by examining the correlations between rs217717 and rs2839698 genotypes and expression levels of H19 mRNA in serum samples from 80 cancer-free controls. Among the 80 cancer-free controls, 37 had the rs2839698 CC genotype, 32 had the CT genotype and 11 had the TT genotype, and the genotype distribution of this SNP was in agreement with the Hardy-Weinberg equilibrium (*P* = 0.345). As shown in Figure 1A, the relative H19 mRNA expression levels were significantly higher for the CT (2.75 ± 0.38), TT (3.60 ± 0.54) and CT+TT genotypes (2.97 ± 0.32) than for the CC genotype (1.61 ± 0.26) (*P* = 0.015, 0.001 and 0.002, respectively). The trend test for the effect of the T allele on the expression was towards significance (*P*_trend_ = 0.003) (Figure 1A). Thirty of the 80 cancer-free controls had the rs217727 CC genotype, 40 had the CT genotype, and 10 had the TT genotype, and the genotype distribution of this SNP was in agreement with the Hardy-Weinberg equilibrium (*P* = 0.551). However, as shown in Figure 1B, the relative H19 mRNA expression levels of the rs217727 CT, TT and CT+TT genotypes were not significantly different from that of the CC genotype (*P* = 0.100, 0.242 and 0.075, respectively). Because rs3741216 A > T and rs3741219 T > C genotypes were not consistently associated with GC risk, their correlations with the related mRNA expression levels were not pursued in further laboratory studies.

### Stratified analysis of polymorphism and GC risk

We performed stratified analyses to evaluate the effects of the variant genotypes on the risk of GC according to age (59 years), sex, smoking status, and residence (Table 4). For the H19 polymorphism rs217727, an elevated risk of GC associated with the variant genotypes was evident in younger subjects (age ≤59) (*P* = 0.033, adjusted OR = 1.52, 95% CI = 1.03-2.22), but not in older subjects (*P* = 0.395, adjusted OR = 1.17, 95% CI = 0.81-1.70). Stratification by smoking status revealed a significant association of rs217727 with GC risk among non-smokers (*P* = 0.013, adjusted OR = 1.47, 95% CI = 1.08-2.00), but not among smokers (*P* = 0.901, adjusted OR = 0.97, 95% CI = 0.58-1.62) (Table 4). There was no significant association between polymorphism and susceptibility to GC in terms of sex or residence.

**Table 4:** Stratified analyses for H19 rs217727 and rs2839698 genotypes in cases and controls

Variable	(CT+TT)/CC for rs217727		Allelic odds ratios and 95% confidence intervals for rs217727		(CT+TT)/CC for rs2839698		Allelic odds ratios and 95% confidence intervals for rs2839698	
	Cases, n (%)	Controls, n (%)	Adjusted OR (95% CI)^a^	*P* value	Cases, n (%)	Controls, n (%)	Adjusted OR (95% CI)^a^	*P* value
Age (y), median								
≤59	182(36.4)/67(13.4)	163(32.6)/91(18.2)	**1.52 (1.03-2.22)**	**0.033**	120(24.0)/129(25.8)	102(20.4)/152(30.4)	1.41 (0.98-2.01)	0.059
＞59	158(31.6)/93(18.6)	144(28.8)/102(20.4)	1.17 (0.81-1.70)	0.395	130(26.0)/121(24.2)	114(22.8)/132(26.4)	1.21 (0.84-1.73)	0.310
Sex								
Females	142(28.4)/69(13.8)	146(29.2)/89(17.8)	1.22 (0.82-1.81)	0.337	98(19.6)/113(22.6)	100(20.0)/135(27.0)	1.20 (0.82-1.75)	0.355
Males	198(39.6)/91(18.2)	161(32.2)/104(20.8)	1.40 (0.98-1.98)	0.064	152(30.4)/137(27.4)	116(23.2)/149(29.8)	**1.43 (1.02-2.00)**	**0.039**
Smoking Status								
Smokers	80(16.0)/44(8.8)	92(18.4)/49(9.8)	0.97 (0.58-1.62)	0.901	69(13.8)/55(11.0)	61(12.2)/80(16.0)	1.56 (0.95-2.56)	0.079
Nonsmokers	260(52.0)/116(23.2)	215(43.0)/144(28.8)	**1.47 (1.08-2.00)**	**0.013**	181(36.2)/195(39.0)	155(31.0)/204(40.8)	1.23 (0.92-1.65)	0.168
Residence								
Rural	199(39.8)/89(17.8)	169(33.8)/95(19.0)	1.26 (0.88-1.80)	0.207	157(31.4)/131(26.2)	105(21.0)/159(31.8)	**1.82 (1.30-2.57)**	**0.001**
Urban	141(28.2)/71(14.2)	138(27.6)/98(19.6)	1.39 (0.94-2.05)	0.097	93(18.6)/119(23.8)	111(22.2)/125(25.0)	0.88 (0.60-1.27)	0.489

The bold in the table indicates statistically significant data.

a Adjusted for age, sex, smoking status, residence, hypertension, and diabetes.

The H19 polymorphism rs2839698 demonstrated a significantly elevated risk associated with this rare genotype in male subjects (*P* = 0.039, adjusted OR = 1.43, 95% CI = 1.02-2.00), but not in female subjects (*P* = 0.355, adjusted OR = 1.20, 95% CI = 0.82-1.75). There was also a significantly increased risk of GC in individuals from rural areas with variant genotypes (*P* = 0.001, adjusted OR = 1.82, 95% CI = 1.30-2.57), but no such association in subjects from urban areas (*P* = 0.489, adjusted OR = 0.88, 95% CI = 0.60-1.27). There was no significant association between the rs2839698 polymorphism and susceptibility to GC in relation to age or smoking status.

There were no significant correlations between the variant genotypes and clinicopathological features of GC, including localization, tumor differentiation, depth of tumor infiltration, and lymph node metastasis (Table 5).

**Table 5:** Associations between variant H19 rs217727 and rs2839698 genotypes and clinicopathologic characteristics of gastric cancer

Variable	(CT + TT) and CC for rs217727		Allelic odds ratios and 95% confidence intervals for rs217727		(CT + TT) and CC for rs2839698		Allelic odds ratios and 95% confidence intervals for rs2839698	
	CT + TT, N	CC, N	Adjusted OR (95% CI)^a^	*P* value	CT + TT, N	CC, N	Adjusted OR (95% CI)^a^	*P* value
Tumor differentiation								
Well	15	5	1.00		14	6	1.00	
Moderate	86	37	0.95 (0.31-2.94)	0.926	64	59	0.41 (0.14-1.18)	0.098
Poor	239	118	0.70 (0.25-1.99)	0.502	172	185	0.38 (0.14-1.03)	0.058
Depth of tumor infiltration								
T1	68	24	1.00		47	45	1.00	
T2	43	15	0.97 (0.44-2.13)	0.935	23	35	0.61 (0.30-1.24)	0.171
T3	110	48	0.76 (0.42-1.37)	0.356	80	78	0.98 (0.58-1.67)	0.955
T4	119	73	0.63 (0.36-1.11)	0.109	100	92	1.00 (0.60-1.66)	0.997
Lymph node metastasis								
Negative	121	49	1.00		88	82	1.00	
Positive	219	111	0.80 (0.53-1.20)	0.277	162	168	0.91 (0.63-1.33)	0.633
Localization								
Cardia	152	69	1.00		116	105	1.00	
Noncardia	188	91	0.91 (0.62-1.34)	0.648	134	145	0.89 (0.62-1.28)	0.527

a Adjusted for age, sex, smoking status, residence, hypertension, and diabetes.

## DISCUSSION

To the best of our knowledge, this study provides the first investigation into associations between H19 polymorphisms (rs217727, rs2839698, rs3741216, and rs3741219) and GC susceptibility in a Chinese Han population. Both variant rs217727T (CT/TT) and rs2839698T alleles (CT/TT) were associated with a significantly increased risk of GC, and the combination of rs217727 and rs2839698 further increased the risk of GC. Using Power and Sample Size Calculation (PS, version 3.0, 2009, http://biostat.mc.vanderbilt.edu/twiki/bin/view/Main/PowerSampleSize), considering H19 rs217727 C > T and rs2839698 C > T mutant alleles in the control group, OR, GC samples, and control sample sizes, the power of our analysis (α=0.05) was 0.862 and 0.854 in 500 gastric cancer cases and 500 controls with adjusted OR = 1.48 and 1.52 for H19 rs217727 C > T and rs2839698 C > T, respectively. These results confirmed that the variant T allele of rs217727 and mutant T allele of 2839698 may be risk factors for GC.

Our data showed that the increased risk of GC associated with the variant genotypes of rs217727 was more pronounced in younger subjects (age ≤59 years) than older subjects. This may be related to accumulated exposure to environmental carcinogens and a weaker immune system in older individuals [27], but more studies are needed to clarify the mechanisms underlying the interaction between H19 polymorphisms and age. Similarly, analyses stratified by smoking status identified a significant association between polymorphism status in nonsmokers, but not in smokers. Tobacco smoking is an accepted independent risk factor for GC [2, 28]. It is possible that the association between the rs217727 polymorphism and GC risk could be masked by accumulated exposure to tobacco carcinogens in smokers, making the association more evident in non-smokers. We also noted that the elevated risk of GC associated with the variant genotypes of 2839698 was more evident in males and in subjects from rural areas. A previous study reported that noncardia cancer was more common in males than females, by a ratio of approximately 2:1, and gastric cardia cancer also had a male-to-female ratio of nearly 4.1:1 in a Chinese population [29]. Our data suggest that the rs2839698 polymorphism may play an important role in men with GC. It has been suggested that genetic differences have their strongest effects under conditions of low environmental pollution [27, 30]. Our results support the hypothesis that the genetic effects might be more pronounced in less-polluted, rural areas [27]. However, further studies are needed to verify these results.

H19 is a lncRNA that can generate a 2.3-kb non-protein coding molecule. Previous studies demonstrated that H19 was highly up-regulated in a variety of tumors, including liver, lung, esophageal, bladder and gastric cancers [22, 23, 25, 26, 31]. H19 thus plays the role of an oncogene. Li et al. reported that, in addition to H19 directly binding to ISM1, it can also encode miR-675, which targets CALN1, and thus promotes cell proliferation, migration, invasion and metastasis in GC [31]. H19 can also promote cell invasion and migration in pancreatic ductal adenocarcinoma, at least partially by increasing HMGA2-mediated epithelial-mesenchymal transition through antagonizing let-7 [32]. Verhaegh et al. reported that a genetic variant of H19 was associated with a decreased risk of bladder cancer in European Caucasians [33]. However, the association between H19 genetic variation and risk of cancer in the Chinese Han population has not previously been reported.

In the current study, H19 rs217727 and rs2839698 were associated with an increased GC risk with borderline significance in the Chinese Han population. Furthermore, variant genotypes of rs2839698 were associated with increased serum mRNA expression levels of H19 in cancer-free controls, suggesting a potential functional impact of this promoter SNP on mRNA levels, thus supporting a role in the susceptibility to GC. The C/T polymorphism rs2839689 is located in 3′untranslated region (UTR) of the H19 gene. Using the software (http://bioinfo.life.hust.edu.cn/lncRNASNP/), we predicted that the conversion of C > T in the 3′UTR rs2839689 polymorphism may create hsa-miR-1285-3p, hsa-miR-3187-5p, hsa-miR-5189-5p, hsa-miR-612, and hsa-miR-6860 and destroy hsa-miR-24-1-5p, hsa-miR-4486 and hsa-miR-566 microRNA (miRNA) binding sites on H19, resulting in gain and loss of function of miRNA-lncRNA interactions. Increasing evidence shows that lncRNAs can be directly regulated by miRNAs, and the influence of rs2839689 may change the structure of H19 at the miRNA binding sites and ultimately affect their function. Further studies are needed to explore the specific mechanisms. In contrast, no such genotype-phenotype correlation was observed for the other H19 rs217727 SNP. The C/T polymorphism rs217727 is located in exon 5 of the H19 gene. However, no miRNAs that combined with rs217727 were identified using a bioinformatics analysis software program (http://bioinfo.uni-plovdiv.bg/microinspector/). Specific structure is known to determine function, and structural changes are thus likely to influence function. SNPs are the simplest form of DNA variation. They can occur between individuals within a population, and may influence promoter activity (gene expression), mRNA conformation (stability), and translational efficiency [34]. Although the rs217727 polymorphism(C > T) did not affect H19 mRNA expression levels, mutation may alter the translational efficiency, potentially leading to alterations in H19 structure, which may ultimately influence the function of H19. A previous study indicated that the effect of H19 on gastric cancer was mediated by direct up-regulation of ISM1, which is a binding protein of H19 [31]. We therefore speculate that alterations in H19 structure may affect H19 binding to ISM1. However, the precise mechanism of H19 action remains unclear, and further investigations are required to verify our hypothesis.

The present study had several limitations. Firstly, the small sample size may have led to insufficient statistical power to detect slight effects, and gene-environment interactions may have been underpowered in the stratified analyses. Secondly, because all subjects were enrolled consecutively from hospitals during the same period, selection bias could not be avoided. Nonetheless, the genotype distribution of the controls in our study was compatible with the Hardy-Weinberg expectations. Thirdly, further analyses were prevented by missing clinical information, such as data on alcohol consumption. Fourthly, although *H. pylori* is an independent risk factor for GC, we did not examine this variable because it was unethical to perform a *H. pylori* test in every subject, especially in controls. In addition, demographic and personal information, such as smoking history, was collected by questionnaire, which may have introduced bias and may in turn have led to insufficient statistical power in our stratified analysis of smoking status. Finally, our study was performed in a Chinese population, and caution should be exercised when extrapolating the data to other regions and ethnic groups.

In conclusion, this hospital-based, case-control study showed that two potentially functional H19 SNPs (rs217727 C > T and rs2839698 C > T) were significantly associated with increased risk of GC in the Chinese Han population. Furthermore, these SNPs may have a combined effect on the risk of GC. Our study also suggested that the H19 promoter rs2839698 C > T polymorphism is functional, modulating susceptibility to GC by altering gene expression levels. These results indicate an important role for H19 variants in GC carcinogenesis, though further larger studies in different populations are needed to validate these results.

## MATERIALS AND METHODS

### Ethics statement

This study was approved by the Ethics Committee of the First Affiliated Hospital of Nanjing Medical University and written informed consent was obtained from all the subjects.

### Subjects

This hospital-based, case-control study included 500 patients with GC and 500 cancer-free controls. All subjects were consecutively recruited from the First Affiliated Hospital of Nanjing Medical University between 2009 and 2014, and all patients were diagnosed with histopathologically-confirmed GC. The exclusion criteria were previous history of: cancer; any metastasized cancer; radiotherapy or chemotherapy; and non-self blood transfusion. Cancer-free controls were randomly selected from the Department of General Surgery during the same period. The controls had no self-reported history of malignancies, and were matched to the cases in terms of age (±5 years) and sex. Both case and control subjects were unrelated, ethnically-Han Chinese individuals from Jiangsu Province or its surrounding regions. Each patient donated 5 ml venous blood after providing written informed consent. A structured questionnaire was used to retrieve information on the subjects, such as age, sex, hypertension, diabetes, smoking history and residence. The questionnaires were administered by trained interviewers who were not aware of the study hypothesis. The following additional parameters were obtained from the medical records of the GC patients: tumor location, histopathological grade, depth of tumor invasion, and lymph node metastasis. According to the pathological reports, 92, 58, 158 and 192 patients were T1, T2, T3 and T4, respectively, and 20, 123 and 357 were well, moderately and poorly differentiated, respectively. Lymph node metastasis was positive in 330 cases and negative in 170. There were 221 and 279 patients with adenocarcinoma of the gastric cardia and non-cardia, respectively.

Subjects were divided into non-smokers and smokers. Individuals who formerly or currently smoked ≥10 cigarettes per day for at least 2 years were defined as smokers. Individuals who had persistent systolic blood pressure >140 mm Hg and diastolic blood pressure >90 mm Hg and/or were currently receiving anti-hypertensive treatment were defined as hypertensive. Subjects with a fasting plasma glucose ≥7 mmol/L or random plasma glucose ≥11 mmol/L, those who showed classic symptoms of hyperglycemia (polyuria, polydipsia, and weight loss), or those requiring insulin or oral hypoglycemic agents were defined as diabetic. Rural or urban residence was determined according to the addresses of the subjects and data were collected by questionnaire.

### SNP selection

We searched tag SNPs in lncRNA H19 in the chromosomal region 11p15.5 using UCSC (http://genome.ucsc.edu/) with the selection criterion of a minor allele frequency >0.05 in the Chinese Han population. Finally, four tag SNPs were identified: rs217727, rs2839698, rs3741216, and rs3741219.

### Genotyping

Genomic DNA was extracted from peripheral blood leukocytes using standard techniques, as described previously [27]. All the genotypes of the four SNPs (i.e., rs217727, rs2839698, rs3741216, and rs3741219) were acquired using the TaqMan-MGB method (Applied Biosystems, Foster City, CA, USA), using two allele-specific TaqMan MGB probes and a polymerase chain reaction (PCR) primer pair. The sequences of the primers and probes are summarized in Table 6. The 10-μl reaction mixture contained 10 ng genomic DNA, 5 μl 2×TaqMan Genotyping Master Mix, 0.25 μl primer, 0.125 μl probe, and 2.5 μl double distilled water. Amplification was performed under the following conditions: 95°C for 10 min followed by 40 cycles of 95°C for 15 s, and 60°C for 1 min. Following the manufacturer's instructions, amplifications were conducted using the 96-well ABI StepOnePlus Real-Time PCR System (Applied Biosystems), and allelic discrimination was performed using SDS 2.4 software (Applied Biosystems). To ensure the accuracy of the genotyping, two negative experimental controls (water) and two positive experimental controls with known genotype were included in each reaction plate. The call rate for the SNPs was 100%. In addition, approximately 10% of the samples were randomly selected and subjected to repeated assays. The final concordance rate for these quality control samples was 100%.

**Table 6:** Information of primers and probes

SNPs	Primer sequence(5′-3′)	Probe sequence
rs217727	F-CAAAGAGACAGAAGGATGAAAAAGAA	G: FAM-TCAACCGTCCGCCG-MGB
C > T	R-CGGCGACTCCATCTTCATG	A: HEX-TCAACCGTCCACCGC-MGB
rs2839698	F-CATCGTCCCCAGCTGATGTC	G: FAM-CTGGGCGCCTACT-MGB
C > T	R-GGAGTGATGACGGGTGGAG	A: HEX-CCTGGGCACCTAC-MGB
rs3741216	F-GCCTCCACGACTCTGTTTCC	T: FAM-CCCTTCTGAATTTTAT-MGB
A > T	R-CACAACTCCAACCAGTGCAAA	A: HEX-CCCTTCTGAATTTAAT-MGB
rs3741219	F-CGAGTGTGCGTGAGTGTGAG	C: FAM-AGTGCCTGCGCAGG -MGB
T > C	R-TAATGGAATGCTTGAAGGCTGCTC	T: HEX-AAGTGCCTGTGCAGG-MGB

### Real-time reverse transcription PCR analysis of H19 mRNA expression levels in serum

The expression levels of H19 mRNA were examined by quantitative reverse transcription (RT)-PCR using total RNA isolated from serum from 80 cancer-free controls using Trizol reagent (Invitrogen, Carlsbad, CA, USA). Extracted RNA was reverse transcribed into first-strand cDNA using Primescript RT Reagent (Takara, Japan). The H19 primers used for quantitative real-time PCR were as follows: forward primer 5′-CCCACAACATGAAAGAAATGGTGC-3′ and reverse primer 5′-CACCTTCGAGAGCCGATTCC-3′. β-actin was used as an internal control and amplified with forward primer 5′-AGAAAATCTGGCACCACACC-3′ and reverse primer 5′-TAGCACAGCCTGGATAGCAA-3′. Amplification reactions were performed in a final volume of 10 μl containing 100 ng cDNA, 0.2 μl primers and 5 μl Master mix. The reaction program was set at 95°C for 30 s, followed by 40 cycles at 95°C for 5 s and 60°C for 30 s. Real-time PCR was performed using a StepOnePlus Real-Time PCR System (Applied Biosystems) using FastStart Universal SYBR-Green Master (Takara). All procedures were performed in triplicate. The 2^−ΔΔCT^ method was used to calculate relative expression levels.

### Statistical analysis

All statistical analyses were performed using SPSS 20.0 (SPSS Inc., Chicago, IL, USA). All tests were two-sided and the criterion of statistical significance was set at *P* < 0.05. Differences in demographic characteristics and genotype frequencies of the four SNPs between cases and controls were calculated using Student's *t*-tests (for continuous variables) and χ^2^ tests (for categorical variables).

The Hardy-Weinberg equilibrium was assessed for controls using the goodness-of-χ^2^ test. Associations between the genotypes and alleles and risk of GC were estimated by odds ratios (OR) and 95% confidence intervals (CIs). The crude OR was calculated using the Woolf approximation method and the adjusted OR was evaluated by the unconditional logistic regression method, with adjustments for age, sex, hypertension, diabetes, smoking status, and residence.

## References

[1] Jemal A, Bray F, Center MM, Ferlay J, Ward E, Forman D (2011). Global cancer statistics. CA Cancer J Clin.

[2] Parkin DM, Bray F, Ferlay J, Pisani P (2005). Global cancer statistics, 2002. CA Cancer J Clin.

[3] Correa P (1992). Human gastric carcinogenesis: a multistep and multifactorial process--First American Cancer Society Award Lecture on Cancer Epidemiology and Prevention. Cancer Res.

[4] Wu MS, Chen CJ, Lin JT (2005). Host-environment interactions: their impact on progression from gastric inflammation to carcinogenesis and on development of new approaches to prevent and treat gastric cancer. Cancer Epidemiol Biomarkers Prev.

[5] Wu AH, Crabtree JE, Bernstein L, Hawtin P, Cockburn M, Tseng CC, Forman D (2003). Role of Helicobacter pylori CagA+ strains and risk of adenocarcinoma of the stomach and esophagus. Int J Cancer.

[6] Yang L, Liu D, Liang S, Guo R, Zhang Z, Xu H, Yang C, Zhu Y (2013). Janus kinase 2 polymorphisms are associated with risk in patients with gastric cancer in a Chinese population. PLoS One.

[7] Yang C, Ma X, Liu D, Wang Y, Tang R, Zhu Y, Xu Z, Yang L (2014). Promoter polymorphisms of miR-34b/c are associated with risk of gastric cancer in a Chinese population. Tumour Biol.

[8] Gu H, Yang L, Sun Q, Zhou B, Tang N, Cong R, Zeng Y, Wang B (2008). Gly82Ser polymorphism of the receptor for advanced glycation end products is associated with an increased risk of gastric cancer in a Chinese population. Clin Cancer Res.

[9] Gu H, Yang L, Tang N, Zhou B, Zhu H, Sun Q, Cong R, Wang B (2008). Association of endothelin-converting enzyme-1b C-338A polymorphism with gastric cancer risk: a case-control study. Eur J Cancer.

[10] Wang KC, Chang HY (2011). Molecular mechanisms of long noncoding RNAs. Mol Cell.

[11] Tsai MC, Spitale RC, Chang HY (2011). Long intergenic noncoding RNAs: new links in cancer progression. Cancer Res.

[12] Gupta RA, Shah N, Wang KC, Kim J, Horlings HM, Wong DJ, Tsai MC, Hung T, Argani P, Rinn JL, Wang Y, Brzoska P, Kong B, Li R, West RB, van de Vijver MJ (2010). Long non-coding RNA HOTAIR reprograms chromatin state to promote cancer metastasis. Nature.

[13] Tsai MC, Manor O, Wan Y, Mosammaparast N, Wang JK, Lan F, Shi Y, Segal E, Chang HY (2010). Long noncoding RNA as modular scaffold of histone modification complexes. Science.

[14] Reik W, Walter J (2001). Genomic imprinting: parental influence on the genome. Nat Rev Genet.

[15] Takai D, Gonzales FA, Tsai YC, Thayer MJ, Jones PA (2001). Large scale mapping of methylcytosines in CTCF-binding sites in the human H19 promoter and aberrant hypomethylation in human bladder cancer. Hum Mol Genet.

[16] Gabory A, Jammes H, Dandolo L (2010). The H19 locus: role of an imprinted non-coding RNA in growth and development. Bioessays.

[17] Gabory A, Ripoche MA, Le Digarcher A, Watrin F, Ziyyat A, Forne T, Jammes H, Ainscough JF, Surani MA, Journot L, Dandolo L (2009). H19 acts as a trans regulator of the imprinted gene network controlling growth in mice. Development.

[18] Brannan CI, Dees EC, Ingram RS, Tilghman SM (1990). The product of the H19 gene may function as an RNA. Mol Cell Biol.

[19] Kallen AN, Zhou XB, Xu J, Qiao C, Ma J, Yan L, Lu L, Liu C, Yi JS, Zhang H, Min W, Bennett AM, Gregory RI, Ding Y, Huang Y (2013). The imprinted H19 lncRNA antagonizes let-7 microRNAs. Mol Cell.

[20] Thorvaldsen JL, Duran KL, Bartolomei MS (1998). Deletion of the H19 differentially methylated domain results in loss of imprinted expression of H19 and Igf2. Genes Dev.

[21] Adriaenssens E, Dumont L, Lottin S, Bolle D, Lepretre A, Delobelle A, Bouali F, Dugimont T, Coll J, Curgy JJ (1998). H19 overexpression in breast adenocarcinoma stromal cells is associated with tumor values and steroid receptor status but independent of p53 and Ki-67 expression. Am J Pathol.

[22] Zhang L, Yang F, Yuan JH, Yuan SX, Zhou WP, Huo XS, Xu D, Bi HS, Wang F, Sun SH (2013). Epigenetic activation of the MiR-200 family contributes to H19-mediated metastasis suppression in hepatocellular carcinoma. Carcinogenesis.

[23] Kondo M, Suzuki H, Ueda R, Osada H, Takagi K, Takahashi T (1995). Frequent loss of imprinting of the H19 gene is often associated with its overexpression in human lung cancers. Oncogene.

[24] Douc-Rasy S, Barrois M, Fogel S, Ahomadegbe JC, Stehelin D, Coll J, Riou G (1996). High incidence of loss of heterozygosity and abnormal imprinting of H19 and IGF2 genes in invasive cervical carcinomas. Uncoupling of H19 and IGF2 expression and biallelic hypomethylation of H19. Oncogene.

[25] Hibi K, Nakamura H, Hirai A, Fujikake Y, Kasai Y, Akiyama S, Ito K, Takagi H (1996). Loss of H19 imprinting in esophageal cancer. Cancer Res.

[26] Luo M, Li Z, Wang W, Zeng Y, Liu Z, Qiu J (2013). Upregulated H19 contributes to bladder cancer cell proliferation by regulating ID2 expression. FEBS J.

[27] Zhu H, Yang L, Zhou B, Yu R, Tang N, Wang B (2006). Myeloperoxidase G-463A polymorphism and the risk of gastric cancer: a case-control study. Carcinogenesis.

[28] Gammon MD, Schoenberg JB, Ahsan H, Risch HA, Vaughan TL, Chow WH, Rotterdam H, West AB, Dubrow R, Stanford JL, Mayne ST, Farrow DC, Niwa S, Blot WJ, Fraumeni JF (1997). Tobacco, alcohol, and socioeconomic status and adenocarcinomas of the esophagus and gastric cardia. J Natl Cancer Inst.

[29] Kelley JR, Duggan JM (2003). Gastric cancer epidemiology and risk factors. J Clin Epidemiol.

[30] Hung RJ, Boffetta P, Brennan P, Malaveille C, Gelatti U, Placidi D, Carta A, Hautefeuille A, Porru S (2004). Genetic polymorphisms of MPO, COMT, MnSOD, NQO1, interactions with environmental exposures and bladder cancer risk. Carcinogenesis.

[31] Li H, Yu B, Li J, Su L, Yan M, Zhu Z, Liu B (2014). Overexpression of lncRNA H19 enhances carcinogenesis and metastasis of gastric cancer. Oncotarget.

[32] Ma C, Nong K, Zhu H, Wang W, Huang X, Yuan Z, Ai K (2014). H19 promotes pancreatic cancer metastasis by derepressing let-7's suppression on its target HMGA2-mediated EMT. Tumour Biol.

[33] Verhaegh GW, Verkleij L, Vermeulen SH, den Heijer M, Witjes JA, Kiemeney LA (2008). Polymorphisms in the H19 gene and the risk of bladder cancer. Eur Urol.

[34] Shastry BS (2009). SNPs: impact on gene function and phenotype. Methods Mol Biol.

